# Characterizing Olive Grove Canopies by Means of Ground-Based Hemispherical Photography and Spaceborne RADAR Data

**DOI:** 10.3390/s100807476

**Published:** 2011-07-28

**Authors:** Iñigo Molina, Carmen Morillo, Eduardo García-Meléndez, Rafael Guadalupe, Maria Isabel Roman

**Affiliations:** 1 ETSITGC, Technical University of Madrid, Campus SUR, Ctra. de Valencia, km.7, Madrid 28031, Spain; E-Mails: mariadelcarmen.morillo@upm.es (C.M.); rafael.guadalupe@upm.es (R.G.); maisa.romandiaz@gmail.com (M.I.R.); 2 Facultad de Ciencias Biológicas y Ambientales, Area de Geodinámica Externa, Campus de Vegazana, Universidad de León, León 24071, Spain; E-Mail: egarm@unileon.es

**Keywords:** active remote sensing, RADARSAT 2, soil roughness, soil moisture, backscattering coefficient, microwave scattering models, hemispherical photography, Gap Fraction, Leaf Area Index (LAI)

## Abstract

One of the main strengths of active microwave remote sensing, in relation to frequency, is its capacity to penetrate vegetation canopies and reach the ground surface, so that information can be drawn about the vegetation and hydrological properties of the soil surface. All this information is gathered in the so called backscattering coefficient (σ^0^). The subject of this research have been olive groves canopies, where which types of canopy biophysical variables can be derived by a specific optical sensor and then integrated into microwave scattering models has been investigated. This has been undertaken by means of hemispherical photographs and gap fraction procedures. Then, variables such as effective and true Leaf Area Indices have been estimated. Then, in order to characterize this kind of vegetation canopy, two models based on Radiative Transfer theory have been applied and analyzed. First, a generalized two layer geometry model made up of homogeneous layers of soil and vegetation has been considered. Then, a modified version of the Xu and Steven Water Cloud Model has been assessed integrating the canopy biophysical variables derived by the suggested optical procedure. The backscattering coefficients at various polarized channels have been acquired from RADARSAT 2 (C-band), with 38.5° incidence angle at the scene center. For the soil simulation, the best results have been reached using a Dubois scattering model and the VV polarized channel (r^2^ = 0.88). In turn, when effective LAI (LAI_eff_) has been taken into account, the parameters of the scattering canopy model are better estimated (r^2^ = 0.89). Additionally, an inversion procedure of the vegetation microwave model with the adjusted parameters has been undertaken, where the biophysical values of the canopy retrieved by this methodology fit properly with field measured values.

## 1. Introduction

Microwave remote sensing has increasingly been used in the Earth Sciences, such as Environment, Hydrology, Agriculture, Forestry, Geology, *etc.*, where these data and associated techniques are able to provide information about biomass, canopy descriptors indices, soil moisture and roughness, as well as some other related variables. Moreover, for a specific vegetation canopy, the common knowledge of specific variables which can be derived from SAR data along a hydrological cycle, allows determining the irrigation status of agricultural crops [1–3].

In the microwave domain, the study of the vegetation canopy requires one to evaluate separately the electromagnetic behavior of soil and vegetation layers, as well as the specific geometric properties of their constituents. Soil surface scattering models need to take into account statistical roughness parameters, such as profile height displacement standard deviation and correlation length, in addition to the dielectric properties that are responsible of the soil reflectivity properties [4,5]. These variables are common hydrological parameters, and their knowledge allows establishing the corresponding surface scattering models, so that they can be further inverted in order to locally or regionally retrieve hydrologic or vegetation related information of the surface [6,7]. A certain number of theoretical and empirical models are available for deriving these values, and they are commonly defined by some specific validity ranges, as it is the case for the Oh and Dubois models [4,8].

Furthermore, for describing the vegetation layer, it is mandatory to define a geometrical model, as well as to define also the electromagnetic properties of its constituents. For this purpose, a certain number of models such as those based on Radiative Transfer (RT) theory have been developed [9–11]. However, the main difficulty is to define appropriate representations, for the geometry and for the electromagnetic properties of the vegetation constituents [12,13]. In order to overcome this difficulty, semi-empirical models have been adopted, since they can be adapted more conveniently to defined scenarios. In this case, the vegetation canopy is usually regarded as a homogeneous or random layer, at a certain height above the terrain surface; this layer is used to compute the attenuation of the wave through this layer [14]. This simplification requires a geometric generalization of the vegetation layer and its constituents. The main simulation models are based on Radiative Transfer theory, which allows for different approaches. In this sense, the ‘Water Cloud Model’ [5], can be efficiently adapted to any vegetated medium, and its constituents can be approximated by more general variables such as the vegetation Water total Content—WTC- [15], or the Leaf Area Index—LAI- of the canopy [16–20]. In the microwave region, it has been proven that the assessment of this variable is also closely related to the operating radar system frequency. As it has been stated in [21], high frequencies are dominated by scattering processes in the crown layer produced by small branches, twigs and foliage within the canopy. LAI is a relevant variable since, besides expressing a biophysical value of the vegetation, it is also considered a descriptor of the leaf geometry and density of the canopy, and therefore it can also be related to the crown or leaf biomass [22–26].

A fundamental aim of this research was to relate vegetation biophysical variables acquired by an optical sensor to radar backscatter. In order to fulfill these prerequisites, it has been considered that C-Band frequency of RADARSAT 2 (5.5 GHz − λ = 5.6 cm) was the most appropriate for studying these processes, due to the similarities of the leaf dimensions of the observed vegetation canopy compared with the Radar system wavelength. In addition, full polarimetric mode has been selected as reference imagery in order to investigate the sensitivities of the different polarized channels to the effect of the canopies geometries. In this respect, a key point of this research is to differentiate between ‘Effective LAI’ and ‘True LAI’, since they express different concepts of leaf distribution within the canopy. Methods for determining these two variables are described by the ‘Gap Fraction Theory’ [27,28], where true and effective LAI’s are related through a clumping index. Moreover, many instruments for the observation of these two variables are available. Among them, the technique of hemispherical photography is very suitable, due to its accuracy, affordable devices and to the fact that green elements of vegetation can be very easily discriminated from trunks, branches, *etc.* [29,30]. Accordingly, this technique has been chosen for this research, integrating the different sets of derived biophysical values into specific vegetation scattering models. Subsequently, an inversion procedure has been tested in order to infer these biophysical variables over larger areas.

This paper is organized as follows. In Section 2, the study area and the motivations that have led to this particular study are described. Additionally, details on the SAR data used for this work are also given, which are followed by a list of field materials and methods employed in this research. The scattering models used in this work for extracting and processing physical and biophysical variables of the canopy, are also presented there. Then, the results reached at each stage of this research are presented and discussed (Section 3). Finally, some conclusions are drawn from the suggested methodology and achieved results.

## 2. Materials and Methods

### 2.1. Study Area

**Figure 1.**
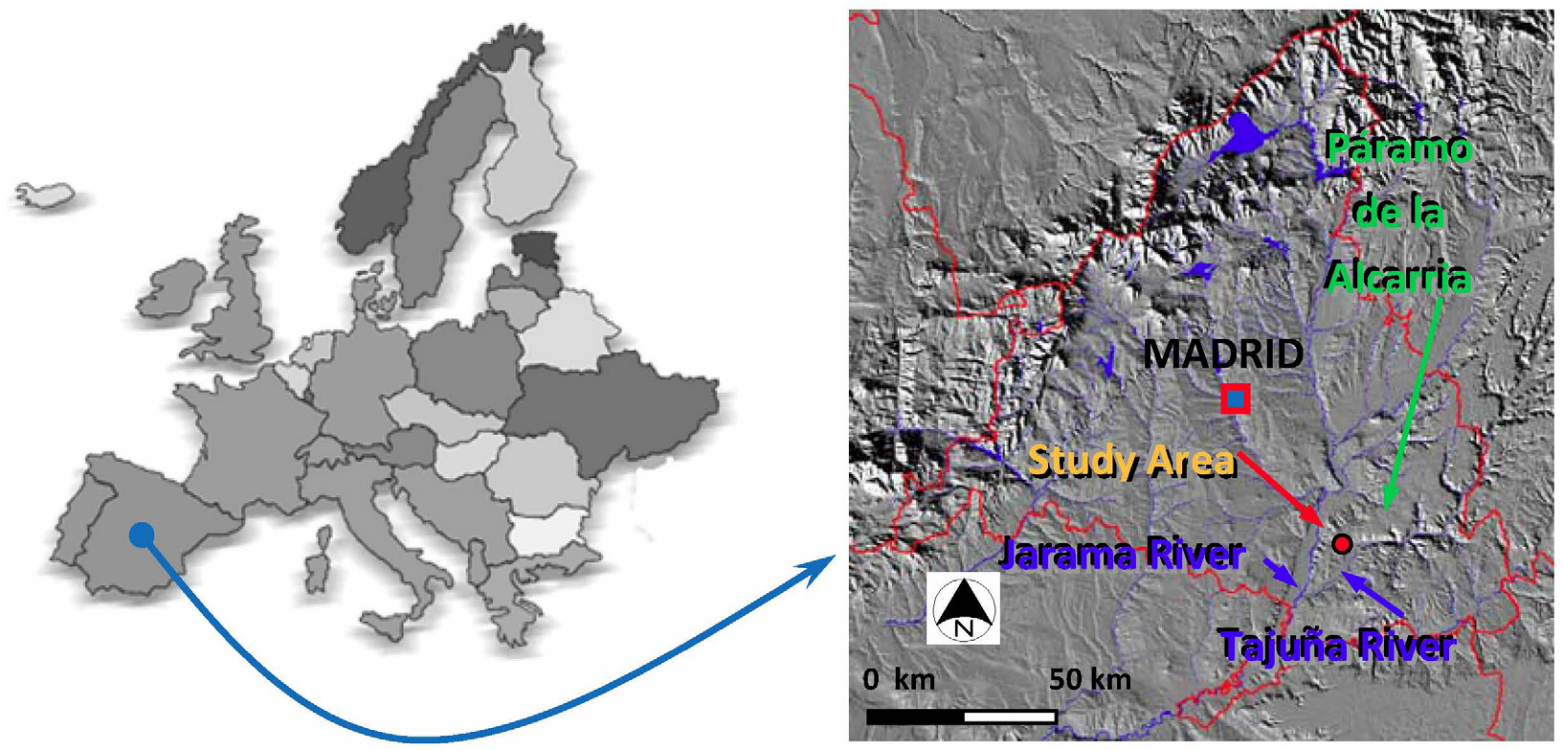
Geographical situation of the study area.

The research area is located in central Spain, near the city of Madrid, on the Southern part of a geomorphologic formation named ‘Páramo de la Alcarria’, demarcated by the Tajuña and Jarama rivers basins (Figure 1). Two main stratigraphic domains are differentiated on this formation, belonging respectively to a Neogene and Quaternary formation, which are usually formed by limestones, tobaceous limestones, marls, clays, sandstones, and conglomerates. The most frequent soils in this area are Inceptisols, formed by weathering of the parent material, in this case limestones. The second soil type in this area is made up of Entisols. According to the agroclimatic classification of Papadakis, this area is classified as a Mediterranean-Temperate domain with continental influences, characterized by remarkable annual thermal amplitude with a marked summer drought. Common annual temperature variations are between 13–14 °C, with a summer average of 22–25 °C, and a winter average of near 6 °C. During the months of July and August the mean temperature exceeds frequently 30 °C.

**Figure 2.**
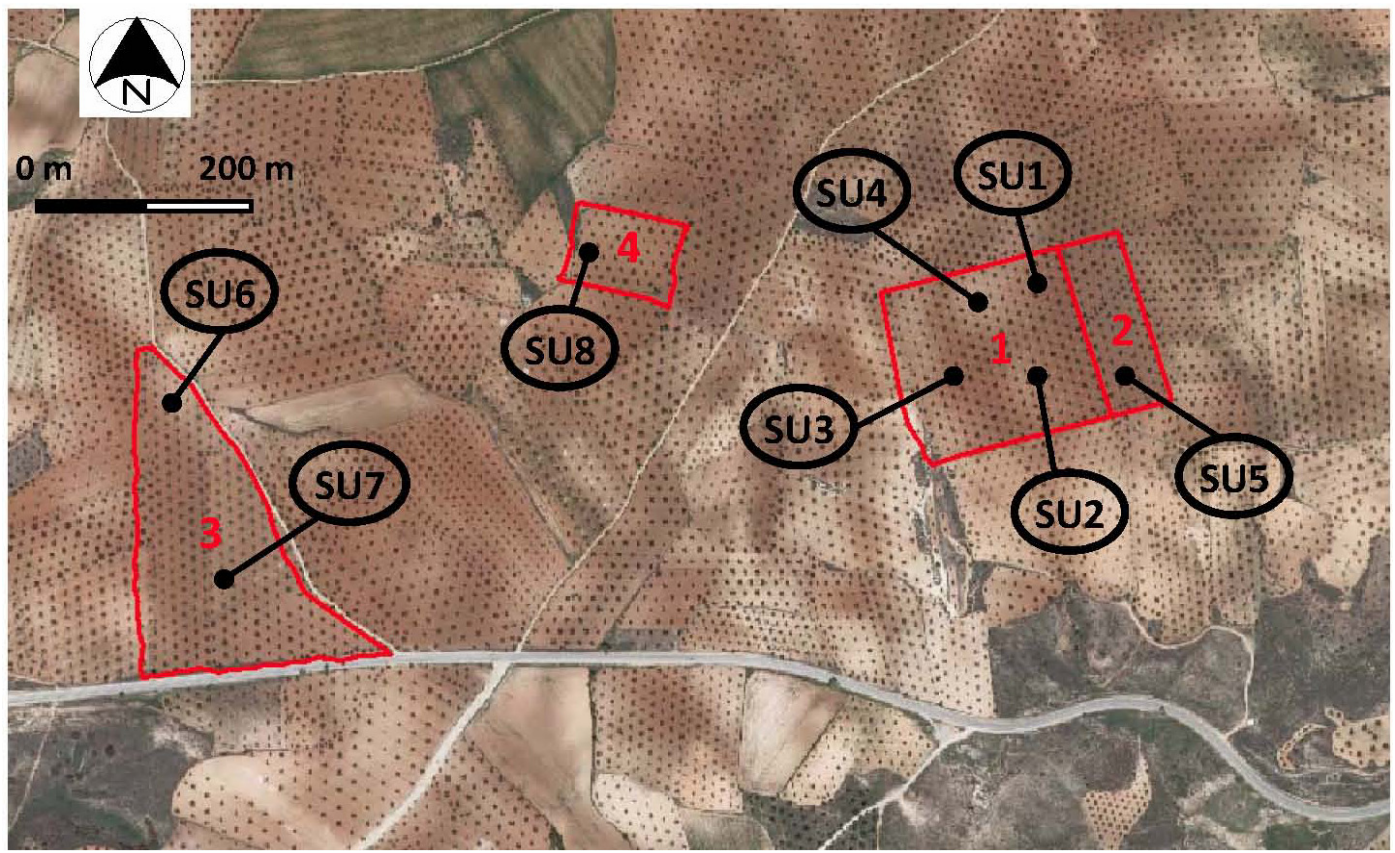
Location of olive grove study areas and corresponding sampling units (SU).

The most abundant landcover and landuse types in this region can be classified into agricultural land, with irrigated crops (corn, alfalfa, *etc.*), and rainfed crops such as barley, olive groves, vineyards, *etc.* In the case of olive groves, they are clearly distributed into homogeneous parcels throughout the study area (Figure 2).

Natural land is generally made up of typical Mediterranean vegetation like shrubs and bushes, as well as Mediterranean oaks. Large coniferous extensions of *Pinus halepensis* and *Pinus pinea* are also present. From these vegetation typologies, olive groves have been chosen for this research for different reasons. First, this landcover is not only referred to as a local representative landscape, but it also constitutes an essential part of ecological and landscape values in Mediterranean areas. These have been declared as biosphere zones of the utmost importance, due to their shield effect on the underlying soils, its biodiversity richness, as well as to the sustainability of the agricultural practices. Additionally, the socioeconomic and cultural significance that this landuse represents for the different Mediterranean countries must also be mentioned [31]. Moreover, in the field of microwave observation systems, this cover type has not been frequently analyzed in the last decade; the most representative works are described in [3,32,33].

In this respect, the performances of new SAR systems must then be verified. For this purpose, within a large olive groves area, eight sampling units (SU) of approximatively 50 × 50 m, have been identified in order to retrieve hydrological and biophysical vegetation canopies reference values (Figure 2).

### 2.2. SAR Data

**Figure 3.**
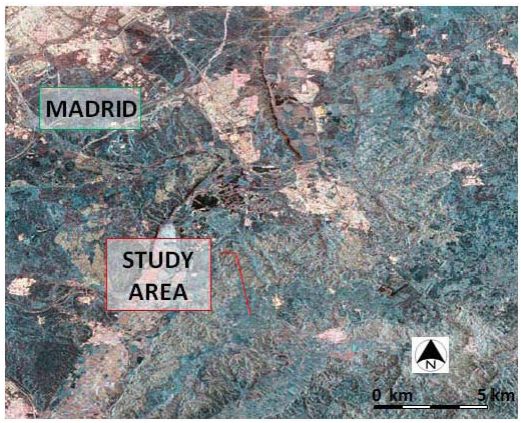
RADARSAT 2 image, PAULI combination (B- |*S_HH_* + *S_VV_*|, G- |*S_HV_*| and R- |*S_HH_* − *S_VV_*|).

The SAR image used in this research has been acquired by the system onboard RADARSAT 2 which operates in full polarimetric mode, with three polarization states (HH, HV y VV) available; these have been processed as a single look complex (SLC) product. Given the specific pattern and architectural properties of olive groves, and the different vegetation constituent’s dimensions, the microwave frequency of this system (5.4 GHz—C Band), as well as its resolution and polarimetric capabilities, appear to be suitable for the aims of this research. The acquisition of this image has previously been programmed, so that soil field surveys and SAR image are temporally coincident. In order to have optimal climatic and soil moisture conditions, the early days of autumn have been chosen for this purpose. Satellite data have been finally acquired on 9 October 2008, with an incidence angle of 38.5° at the scene centre and a nominal resolution of 8 m in range and azimuth. Figure 3 shows a *Pauli* color combination derived from the three polarization states, where the study area is also indicated.

The available polarization states have been filtered with a ‘Lee speckle filter’ with a window width of 5 × 5 cells. Then the backscattering coefficients have been derived from the scattering matrix *S_pq_*. A geometrical transformation has also been applied, so that position of field survey samples and image coordinates may coincide properly. GPS measurements and 1/5,000 scale digital cartography have also been used for refinement of the image geometry. Olive groves areas are distributed spatially over a nearly flat terrain, assuming for this reason, that the terrain effects on these SAR data are negligible.

### 2.3. Physical and Biophysical Canopy Parameters Determination

Soil and vegetation measurements have been driven on the 8 sampling units (SU) introduced in Section 2.1, where the corresponding physical and biophysical parameters were respectively measured by a specific method and instrument.

#### 2.3.1. Soil Physical Parameters

Soil physical parameters comprise both, surface roughness and moisture content measurements. Each roughness measure has systematically been along a direction parallel to the nominal flying line of the satellite. A one meter length 5 × 5 cm gridded tablet has been employed for this task. Once it has been inserted into the soil surface, leveled and oriented, a picture has been recorded with a digital photographic device, so that this original position might be geometrically restored and the profile digitized by means of image processing techniques. This method enables to derive the statistical parameters of the soil surface such as the standard deviations of height displacements (σ) and correlation lengths (*l_c_*) associated to the correlation function of each profile. Subsequently, soil profiles have been obtained by means of a semi-automatic procedure combining image segmentation techniques and manual editing, so that incorrect identified height displacements might be manually corrected.

Soil moisture sampling has been carried out by means of a metallic cylinder of 5 × 5 cm in diameter and in height, which has been in turn inserted into the first 5 cm of the soil surface. The extracted samples were held in a plastic bag with hermetic closure, and subsequently brought to the laboratory where they were weighted and dried in oven at 60 °C for 70 h. Then, each sample has been weighted again. Finally, 14 of these samples have been retained for successive texture analysis. All these samples have been used to derive soil moisture parameters such as gravimetric moisture, bulk density of the material and volumetric moisture. The latter, together with soil texture information have been finally used to compute the soil dielectric constant by means of the Dobson-Peplinsky model [34]. Then, these retrieved soil physical values have been used as input variables for simulating the backscattering coefficient (σ^0^) of the observed soil surfaces by means of the models that will be described in Section 2.4.

#### 2.3.2. Vegetation Biophysical Parameters

Vegetation biophysical variables have been derived by means of hemispherical photographs. This process has been undertaken in each sampling unit where soil roughness and moisture have also been determined, so that these new values may be spatially consistent with the previous ones. This technique allows computing variables such as true and effective LAI’s, Average Leaf Angle (ALA) and fraction cover for individual or group of plants. Regarding acquisition conditions, some properties of the instrument and vegetation cover must also fulfilled [29], *i.e.*, good sensor resolution and sensibility, as well as using an extra wide angle objective (fisheye). For this study a CANON EOS 450D camera (12.6 Mpixels sensor resolution) equipped with a SIGMA Circular Fisheye 4.5 mm f2.8 EX DC has been employed. In order to observe the canopy with this camera, a tripod with a 3D head for orienting the optical axis vertically has also been used. Since the vegetation cover is considered ‘high’, *i.e.*, higher than 70 cm, the sensor has been set at 40 cm above the soil surface. Furthermore, in order to ensure a homogeneous capture of all canopy elements, solar lighting conditions must be as diffuse as possible. Usually, cloud cover days are the most appropriate for this purpose, although the early or late hours of the day are also suitable.

**Figure 4.**
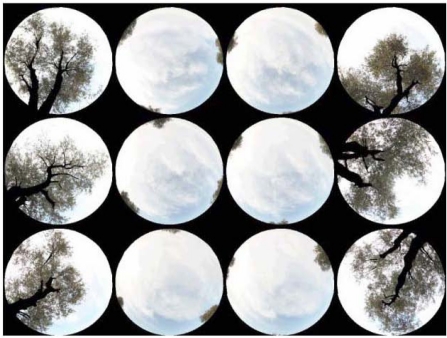
Example of a set of transects describing a sampling unit/vegetation canopy.

The sampling methodology is based on the strategy proposed in [28] and [29], who suggest that for periodic canopies, the acquisition of photographs must be conducted along diagonal transects with equidistant ranges. In this case each transect has an approximated length of 21 m, and each photograph is recorded at equidistant positions of 7 m, resulting thus in four photographs per transect. Each transect is fitted between two single trees. Usually, there are more than one transect per sampling unit, and these are organized along parallel lines, in such a manner that each SU or canopy is described regularly by a set of hemispherical photographs. Figure 4 shows a SU/canopy described with a set of 12 hemispherical photographs taken along three transects.

Each set of photographs has been processed using CAN_EYE software version 1.4 [35], which is able to process up to 20 photographs at the same time. The operating procedure for obtaining the biophysical variables of interest is based on the ‘*Gap fraction*’ Theory described in [27,28]. In the case of a random spatial distribution of infinitely small leaves, is often called transmittance [36]. This quantity also plays an important role in the radiative balance of plants, and is closely linked to the structure of the vegetation canopy. A ray of light crossing a poor developed canopy (which is characterized by low LAI values) will have a high probability of reaching the soil surface; alternatively, if it crosses a denser canopy (high LAI values), this probability will decrease. These concepts can also be used in microwave remote sensing, since such canopies will respectively reveal a high or low transmissivity to the incoming radiation energy. There are several theoretical expressions to relate *Gap Fraction* to the canopy geometry. The most common models [27,28] are based on a random distribution of leaves within the canopy, *i.e.*, leaves are not distributed regularly within the canopy; this variable is referred to as *Effective Leaf Area Index,* and it is related to the *True Leaf Area Index* through the so called clumping index *λ*_0_ [28]. In this sense, Effective LAI is an important variable, as it is also a geometrical or architectural descriptor of the canopy. The method for retrieving biophysical values from this methodology is described in Section 3.1.3.

### 2.4. Microwave Backscattering Models

There are two main theories for modeling vegetation canopies in the microwave domain which take into account soil and vegetation contributions separately, *i.e.*, the Distorted Born Approximation (DBA) and the Radiative Transfer (RT) Theory [37,38]. In this work, the second approach has been adopted. In theoretical scattering RT models, vegetation can be treated as a discrete medium over the soil surface, which in turn is considered as a continuous dielectric surface [5,11,21,39]. RT models can also derive into very sophisticated modeling procedures which take into account 3D simulations of the vegetation canopy constituents for describing radiation interactions between them, as it is the case in [40,41]. In [40] it is also stated that it is possible to use the same structural description of the canopy to drive optical and microwave scattering models. Although, optical radiation models are not addressed in the current study, the basic principle is recovered here, where a more generalized approach has been followed. For this purpose, the structural description of the canopy is represented by Effective LAI (LAI_eff_), which in turn is integrated into a modified version of the so called Water Cloud Model—WCM-developed by [14], which can be made up of explicit physical and biophysical variables of the canopy [42], such as True LAI or Effective LAI, among others. The microwave scattering methods adopted in this work are described in the next sections.

#### 2.4.1. Simulation of the Dielectric Surface

Each microwave scattering model used for describing a vegetation canopy needs to incorporate a simulation of the soil surface beneath the vegetation layer, which is modeled as a dielectric surface. This task can be undertaken by means of many existing models. Amongst these, the Oh and Dubois surface models, referred to as semi-empirical [7] and empirical [8], respectively, might initially appear suitable for the soil surfaces conditions given in the study area. In addition, each of these models has also the capacity to compute at least two polarization states of the backscattering coefficient, which is also important, as the radar data used in this work is a full polarimetric mode. Therefore the suitability of applying both models will depend exclusively on their validity ranges, and the ability of being adapted to the soil conditions of the study sites. In this sense, the Oh model allows computing the three polarizations states of the backscattering coefficient, for soil roughness values −*k* (*k* is the wave number and the height vertical displacements of the soil surface) comprised between 0.1 and 6, and soil volumetric moisture (m_v_) values ranging from 9.5% to 31%. Additional details for this model can be found in [4]. Regarding the Dubois model, this method takes only into account the co-polarized channels, and is limited to *kσ* < 2.5 and m_v_ < 35% soil conditions. The range of incidence angles of the acquired radar image are within the validity ranges for these two models. In the case of the Dubois model, the two polarization states of the backscattering coefficient are approached by:

(1) $$\begin{array}{l} \sigma_{\mathrm{hh}}^{0}=10^{-2.75}\frac{{\text{cos}}^{1.5}\theta_{i}}{{\text{sin}}^{5}\theta_{i}}10^{\left(0.028\varepsilon \text{tan}\theta_{i}\right)}{\left(k\sigma \text{sin}\theta_{i}\right)}^{1.4}\lambda^{0.7}\\ \sigma_{\mathrm{vv}}^{0}=10^{-2.35}\frac{{\text{cos}}^{3}\theta_{i}}{{\text{sin}}^{3}\theta_{i}}10^{\left(0.046\varepsilon \text{tan}\theta_{i}\right)}{\left(k\sigma \text{sin}\theta_{i}\right)}^{1.1}\lambda^{0.7} \end{array}$$

where *λ* is the wavelength of the radar system, and ε the soil dielectric constant. The Dubois model is selected here as it delivers reasonable results in conditions with bare soil or sparse vegetation [42], which matches the conditions seen in this study.

#### 2.4.2. RT Vegetation Scattering Models—WCM

**Figure 5.**
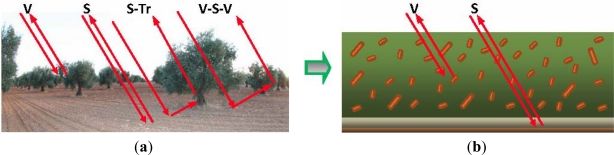
(**a**) Interactions produced between the different constituents/contributions (**‘V’**: vegetation, **‘S’**: soil, **‘Tr’**: trunk); and (**b**) generalization of the vegetation canopy.

Generally, Radiative Transfer models for vegetation canopies take into account contributions of the soil surface (S) and vegetation (V) separately, as well as multiple interactions between them, which are produced between soils, trunks (Tr) and/or primary branches. However, for the sake of simplicity, within the framework of this work, these interactions have not been considered, and as they are not considered to be a dominant term in the copolarized returns [43,44]. Thus, the selected microwave canopy models will consist only of two layers, represented by the contributions of soil and vegetation, which is considered to be formed by a homogeneous layer of particles (Figure 5(b)).

Therefore, according to this principle the total backscattering coefficient will be expressed by:

(2) $$\sigma^{0}=\sigma_{\mathrm{vegetation}}^{0}+\sigma_{\mathrm{soil}}^{0}$$

A first characterization the vegetation canopy has been undertaken using the Rayleigh vegetation backscattering model (Equation (5)). This model appears to be appropriate when the size of the vegetation constituent is comparable to the wavelength of the radar system, and it has already been applied successfully to different types of vegetation covers [39,43,45]. In this case, the radar system operates in C Band (*λ* = 5.6 cm for RADARSAT 2), whose wavelength is similar to the dimensions of the olive tree leaves. This model requires only the soil simulated backscattering coefficient, as well as the respective radar incidence angles for each position on the SAR image. Then, it evaluates the transmissivity of the vegetation layer (Γ) and its albedo (ω) according to the following expression:

(3) $$\sigma_{\mathrm{pp}}^{0}=\underset{\text{vegetation contribution}}{\underset{︸}{0.75\cdot \omega .\text{cos}\theta_{i}\cdot \left(1-\Gamma^{2}\right)}}+\underset{\text{soil contribution}}{\underset{︸}{\Gamma^{2}\cdot \sigma_{\mathrm{pp}\;\mathrm{soil}}^{0}}}$$

where ‘*pp*’ stands for horizontal (*hh*) or vertical (*vv*) polarization. Once Γ and ω are derived, this model might be used for retrieving the soil contribution of the vegetation canopy according to the following expression (Equation (6)):

(4) $$\sigma_{\mathrm{pp}-\mathrm{soil}}^{0}=\frac{\sigma_{\mathrm{pp}}^{0}-0.75\cdot \omega \cdot \text{cos}\theta_{i}\cdot \left(1-\Gamma^{2}\right)}{\Gamma^{2}}$$

For a continuous set of observation incidence angles, this expression enables one to compute a raster layer representing the soil contribution of the observed canopies using known values of transmissivity and albedo. This is also the first step for extracting soil moisture content values (m_v_), which can also be carried by means of Oh and/or Dubois inversion procedures. This process requires that both polarizartion states (*pp = hh* and *pp = vv*) are computed [7,8].

The second model taken into consideration is based on the model specified in [46], which is a modified version of the original Water Cloud Model (WCM). Here, the Leaf Area Index (LAI) is used as a descriptor of the canopy, and is integrated according to:

(5) $$\sigma_{\mathrm{pp}}^{0}=A\cdot \left[1-e^{\left(-2B\cdot \mathrm{LAI}/\text{cos}\theta_{i}\right)}\right]\cdot \text{cos}\theta_{i}+\underset{\mathrm{soil}\;\;\;\mathrm{contribution}\left(\sigma_{s}^{0}\right)}{\underset{︸}{\left(C+D\cdot m_{s}\right)}}\cdot e^{\left(-2B\cdot \mathrm{LAI}/\text{cos}\theta_{i}\right)}$$

where C and D are soil specific parameters which hold a lineal relationship with the soil moisture content m_s_, while the terms A y B are linked to the vegetation canopy and represent respectively the density and attenuation of the vegetation layer [21]. First, this model is solved for parameters C and D using a linear regression, in which soil moisture content values are evaluated against measured backscattering values. Then, A and B parameters are evaluated by means of a non linear regression. In this study, Equation (7) is modified so that the soil contribution is replaced by the soil scattering values derived from soil simulation process, which is the written as:

(6) $$\sigma_{\mathrm{pp}}^{0}=A\cdot \left(1-e^{-2B\cdot \mathrm{LAI}/\text{cos}\theta_{i}}\right)\cdot \text{cos}\theta_{i}+\sigma_{\mathrm{pp}\_\mathrm{soil}}^{0}\cdot \left(e^{-2B\cdot \mathrm{LAI}/\text{cos}\theta_{i}}\right)$$

This second model has been selected and further adapted, due to its convenience for integrating the physical and biophysical variables considered in this study, *i.e.*, True LAI and Effective LAI. Like the Rayleigh model, Equation (6) can also be inverted in order to derive the soil contribution for both polarized returns, so that soil moisture might in turn be retrieved. Theoretically, soil contributions derived from these two canopy approximations are considered to be consistent, *i.e.,* both methods must deliver equivalent results for the soil surface, as they are computed under identical spatial and temporal conditions for the same canopy type, using the same calibration data. The main difference between both models is that Equation (6) adds an explicit vegetation descriptor of the canopy for calibrating its parameters, e.g., LAI, while in Equation (3) the latter might be implicitly contained into parameters Γ and ω. Instead, in this work and in the manner described in [46], once the parameters of Equation (6) are derived, the possibility of generating a map of LAI values by inverting this equation has been explored, where the original soil contribution (*C* + *D* · *m_s_*) is again substituted by a set of soil simulated values ( $\sigma_{\mathrm{pp}-\mathrm{soil}}^{0}$). Such inversion procedure is expressed by:

(7) $$\mathrm{LAI}=-\frac{\text{cos}\theta_{i}}{2B}-\text{ln}\left(\frac{\sigma_{\mathrm{pp}}^{0}-A\cdot \text{cos}\theta_{i}}{\sigma_{\mathrm{pp}-\mathrm{soil}}^{0}-A\cdot \text{cos}\theta_{i}}\right)$$

For this purpose, it is suggested to take as soil contribution a soil raster layer derived by means of the inverted model represented by Equation (4). This operation will be described in Sections 3.5.1 and 3.5.2.

## 3. Results and Discussion

In this section, the results achieved for soil physical and vegetation biophysical values are first presented. Then, the simulation of the backscattering coefficient by means of soil and vegetation microwave scattering models is also described and analyzed. Finally, LAI values are inferred from an inversion procedure and compared to field reference values.

### 3.1. Soil Surface Roughness Results

**Figure 6.**
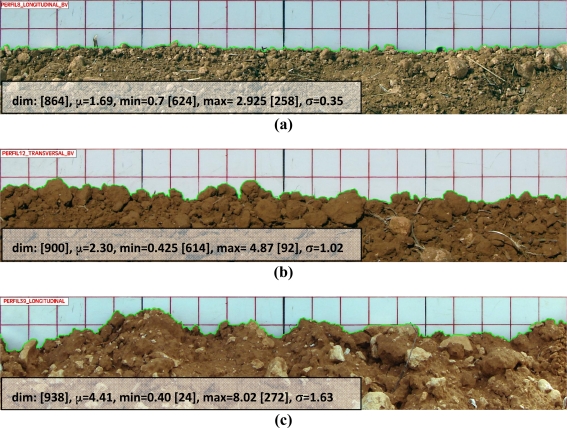
Example of soil roughness profiles (in green) and associated parameters (‘**dim**’: n° of points in the profile, ‘**min**’: minimum vertical displacement in cm, ‘**max**’: max. vertical displ. in cm, ‘**σ**’: height displ. standard deviation in cm), (**a**) smooth surface, (**b**) and (**c**) rough surfaces.

According to the procedure specified in Section 2.3.1, soil surface roughness has been assessed by means of a 5 × 5 cm gridded one meter length plate inserted into the soil surface and recorded on digital pictures. For this purpose, 37 pictures have been registered at singular spatial positions distributed regularly throughout the sample units. Furthermore, these pictures have been processed digitally using a semi-automatic methodology, which enables the acquisition of highly dense profiles describing very precisely the soil surface (green lines in Figure 6).

Each one meter length profile is characterized by at least 400 regularly distributed vertices, whose spatial coordinates allow deriving the vertical displacements of the surface. In turn, these values have been used to compute the standard deviation of these displacements (σ) and the correlation length (*l_c_*). However, the latter was not used, as the scattering models applied in this work do not consider this value.

According to the Rayleigh criterion, for the radar frequency used in this study, surface standard deviations lower than 0.7 cm will be designated as smooth, and conversely rough, for σ higher than this quantity. An example of the surfaces observed in some sampling units is given in Figure 6, which show different degrees of roughness, from smooth to rough. The highest standard deviation was obtained for sampling unit 5 with σ = 2.64 cm, while the lowest was found in sampling unit 3 with σ = 0.32 cm. Although, some of the profiles exhibit low standard deviations, in general most of the surfaces can be considered rough since the mean value is σ = 1.1 cm. The detected differences might be essentially due to the different tillage practices found in each sampling unit.

As a consequence of these results, the gathered soil surface values for the sampling units appear to lie within the validity range of Oh and Dubois models, so they may be integrated into these soil surface scattering simulation models.

### 3.2. Soil Moisture and Texture Results

For soil moisture determination, the results reveal extremely low soil moisture content values. A mean volumetric moisture value of m_v_ = 10% has been reached, with a minimum value of m_v_ = 4.5% and a maximum of m_v_ = 14%. Although, these differences might appear very high, this range of values exhibits unfortunately very low moisture content.

**Figure 7.**
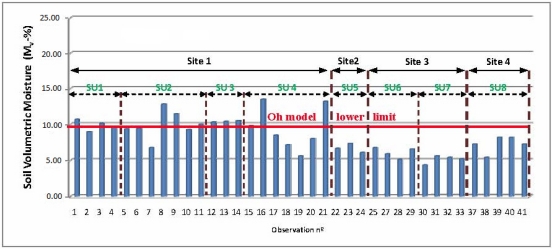
Soil volumetric moisture values for each sampled location.

These variations may be due to errors introduced by the measurement device and method, as well as the spatial distribution of the soil bulk density in the sampling units, which in turn could be produced by the compaction effects resulting from the tillage techniques, as it is stated in [1]. Furthermore, the low values reached for this variable can also be produced by the low precipitations rates during the data acquisition period, combined with a high temperature regime at this time of the year. Figure 7 shows graphically individual values of soil volumetric moisture, where most of these values are observed to be below 10%. While these values are observed to remain within the validity range for the Dubois model (<30% m_v_), most of the moisture observations are far below the lower valid limit for the Oh model, which is therefore considered inappropriate to characterize the soil surface of the current vegetation canopies. In [6], this model is considered that performs correctly for values exceeding 10% of volumetric moisture, which occurs only in a few cases for this soil moisture dataset.

Soil texture knowledge is also important since it determines the properties of water retention and transmission of the soil. Soil textural classes are based on the proportions of sand, silt and clay expressed in percentages. In this work, 14 soil samples have been retained for deriving representative soil texture classes. For those not included in this analysis, their class has been assigned based on the nearest determined texture. For this purpose, a conventional methodology based on Stokes Law has been applied. According to the USDA soil texture classification scheme, almost all analyzed samples belong to class clay, although it has been found than in some sampling units the textural class ‘silty clay’ is also present. Volumetric soil moisture as well as soil texture are needed as input variables for dielectric models. The Dobson-Peplinsky model has been selected and applied in order to compute the complex dielectric constant required for the soil surface models introduced in Section 2.3.1.

### 3.3. Vegetation Biophysical Parameters

**Figure 8.**
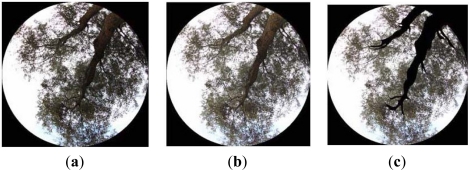
Edition of photographs, (**a**) Original image, (**b**) Gamma correction, (**c**) Elimination of non-green vegetation elements eliminated.

Biophysical values of the vegetation canopy have been retrieved using the methodology depicted in Section 2.3.2. First, in order to minimize optic distortions produced on canopy elements due to far extreme observation angles, it is necessary to eliminate these effects from photographs, which is done by reducing the field of view to from [0°–90°] to [0°–60°]. Moreover, due to illumination conditions, it is not possible to distinguish properly between green and non green elements such as branches, twigs, *etc.* In order to facilitate this discrimination, a contrast correction can be applied [Figure 8(b)], so that these elements are better recognized, and non green elements might be eliminated in order to exclude them during the segmentation process. Then, a manual edition for eliminating non green elements has been carried out [Figure 8(c)].

**Figure 9.**
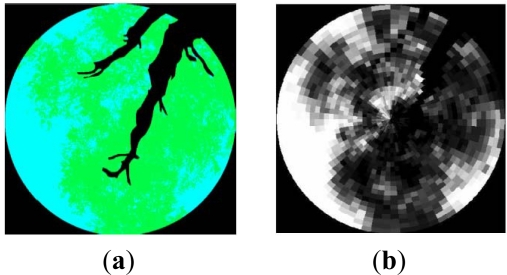
Hemispherical view of a single tree, (**a**) segmented image, (**b**) gap fraction P_0_.

Then a segmentation process must be carried out. This operation is considered to be as the most critical in the extraction chain, since the final result will depend on the approach taken to discriminate between the different classes contained in these pictures [Figure 9(a)]. In this case, the referred classes will only cover sky and green vegetation, as trunks, primary and secondary branches have previously been eliminated. Nevertheless, fine branches and twigs will persist, given the difficulty of separating them from leaves.

**Figure 10.**
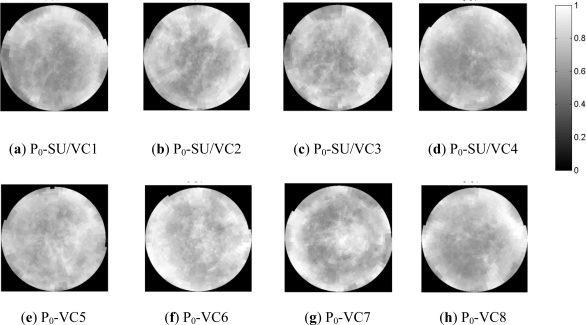
(**a**)–(**h**) Average Gap Fractions (P_0_) of the eight studied sampling units (SU)/vegetation canopies (VC, P0 [0°, 60°]).

The segmentation operation is carried out by means of a supervised training, where image values are assigned to their corresponding class applying the *Convex Hull* algorithm [29]. A segmentation result as well as its corresponding derived gap fraction is illustrated in Figure 9a and 9b, respectively. Then, the distinct average gap fractions (P_0_) for the eight analyzed olive grove canopies (SU) are highlighted in Figure 10.

These average P_0_ representations depict the geometry/architecture of each observed canopy and reveal the grouping (clumping) effect of the vegetation constituents (mainly leaves in this case). Thus, each vegetation cover is supposed to have a specific behavior to the electromagnetic waves. In this sense, lighter grey scales values indicate a greater transmissivity of the vegetation cover, while the darker indicate a higher opacity. This is the rationale for assessing to which extent the grouping of vegetation components, represented by effective LAI, has an influence on the characterization of the backscattering coefficient, in comparison to the regular distribution of leaves described by true LAI.

As a result of these gap fraction representations, the following parameters are derived: Clumping Index (*λ*_0_), Average Leaf Angle (ALA), Effective LAI (LAI_eff_) and True LAI (LAI_true_), as well as LAI_true_ 57° and LAI_eff_ 57°, which are estimated from the gap fraction measured at a zenithal angle of 57.5°. For this particular direction, the projection function G(*θ_v_*,*φ_v_*) can be considered independent from leaf inclination, simplifying therefore the LAI estimation procedure [47].

An additional parameter of interest that must be also mentioned is the canopy or plant cover fraction, which is defined as the fraction of ground covered by vegetation [36]. In spite of the knowledge of all these parameters, in this work, only LAI_eff_ and LAI_true_ have been taken into consideration, leaving the treatment of the remaining parameters for forthcoming studies. The range of values for these two variables varies from 0.28 to 0.47 m^2^.m^−2^ for LAI_eff_ and from 0.47 to 1.5 m^2^.m^−2^ for LAI_true_. For the latter, these values are in accordance with those given in other studies as in [48,49]. Unfortunately, no other reference values for effective LAI have been found in the bibliography for this type of vegetation canopy. The different LAI values for the studied sampling units are summarized in Table 1, where the P_0_ estimated standard deviation (σ_Po_) values are given for three hemispheric angles, *i.e.*, zenith (0°), 57°5 and 60°. These values increase monotonically between the two extreme angles.

### 3.4. Soil Scattering Models

**Figure 11.**
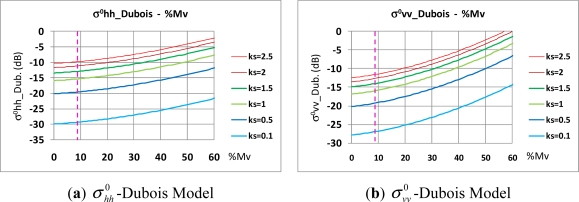
(**a**,**b**) Variations of $\sigma_{\mathrm{pp}}^{0}$ due to soil volumetric moisture according to different soil roughness degrees (ks ≡ kσ), Oh and Dubois Models.

Once the physical values of the soil surface are available, the next phase consists in simulating the soil surface backscattering coefficient. Due to the volumetric soil moisture results derived for the study area only the Dubois model is assessed. First, a sensitivity analysis of the behavior of this model to the range of the sampled moisture and roughness values has been undertaken. For this purpose, a set of plotted curves [Figure 11(a,b)] illustrating the behavior of soil backscattering coefficient to the soil volumetric moisture content for fixed values of surface roughness (*kσ*). This analysis has been performed for the two polarization sates (*pp = hh* and *vv*), and the required angle of incidence has been set up fixed at 38.5°, *i.e.,* the incidence angle at the scene center. Figure 11(a) shows that, for the given ‘*kσ*’ roughness values, $\sigma_{\mathrm{hh}}^{0}$ is not too sensitive to the soil moisture content for m_v_ values near to 10%. In turn, in the domain of low surface roughness values, *i.e.*, *kσ* ∈ [0.1–1], this coefficient is especially sensitive to short variations of this value, while *kσ* tends to saturate for values above 2, so it is possible to appreciate the upper valid limit (*kσ* = 2.5) for this variable and model. This situation is also observed for the vertical case $\sigma_{\mathrm{vv}}^{0}$, though it appears that this coefficient is more sensitive to m_v_ for values near to 10%.

Thus, the outlined model show slight different behaviors depending on the polarization used, which might also produce different results, but in both cases, the Dubois model appears appropriate for the soil simulation, as the existing set of soil physical values fall within the valid limits of this model. Only roughness values approaching *kσ* ∼2.5 are expected to not deliver good results, which occurs only in few cases. In this preliminary analysis and for the sake of simplicity, the backscattering coefficient has been represented in decibels (dB), while the results for the forthcoming simulation process using the existing values of surface roughness soil moisture content (m_v_) will be expressed in m^2^.m^−2^. For this set of values, their corresponding $C-\sigma_{\mathrm{pp}}^{0}$ coefficients have simulated using the Dubois model. In turn, these simulated results have been compared to the image measured backscattering coefficients using a linear statistical regression procedure, where 37 available measured/simulated observations pairs were initially available. Then, the best correlated observations have been compared graphically. As a general rule, all simulated backscattering coefficients must exhibit lower values than those derived from the image, so that the attenuation effect of the vegetation layer on the radiation reaching the soil surface is also considered.

**Figure 12.**
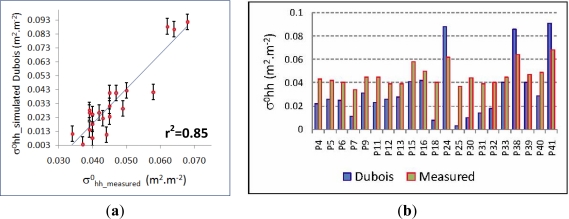
(**a**) Dubois- $\sigma_{\mathrm{hh}}^{0}$ *vs.* measured- $\sigma_{\mathrm{hh}}^{0}$ regression analysis, (**b**) Dubois- $\sigma_{\mathrm{hh}}^{0}$ simulated *vs.* measured- $\sigma_{\mathrm{hh}}^{0}$ values. (Error bars show the standard error value).

For the horizontal polarization state ( $C-\sigma_{\mathrm{hh}}^{0}$), the best regression analysis has been reached with a set of 21 observed values, and with a coefficient of determination r^2^ = 0.85 (Figure 12(a)). With this values, a reasonable trend between backscattering values (simulated *vs.* measured) is observed (Figure 12(b)), although in three particular cases (P24, P38 and P41), the simulated $\sigma_{\mathrm{hh}}^{0}$ exceed their corresponding measured values. This means that the combined effect of the vegetation layer and the soil surface might not be properly modeled. In these three cases, the surface roughness value (*kσ*) is 2.39, which is the highest value in this data set and approximates the upper limit of this variable for the Dubois model. This may then explain the discrepancy between the observed and simulated values. In any case, the reported observations should not be used for subsequent operations, which imply that in the horizontal case, the dataset of surface physical surface values for modeling the vegetation canopies will not be sufficient for subsequent simulations and statistical analysis.

**Figure 13.**
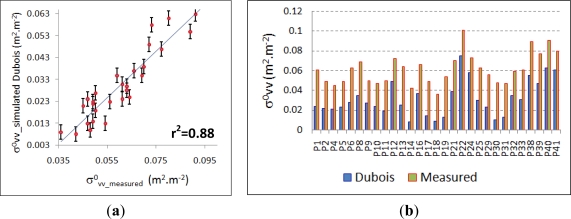
(**a**) Dubois- $\sigma_{\mathrm{vv}}^{0}$ *vs.* measured- $\sigma_{\mathrm{vv}}^{0}$ regression analysis, (**b**) Dubois- $\sigma_{\mathrm{vv}}^{0}$ simulated *vs.* measured- $\sigma_{\mathrm{vv}}^{0}$ values. (Error bars show the standard error value)

In turn, for the horizontal polarization state ( $C-\sigma_{\mathrm{vv}}^{0}$), good results are achieved using the results of the soil simulation processed and the image measured backscattering values. In this case, the coefficient of determination reaches r^2^ = 0.88 (Figure 13(a)) with a set of 29 observed values. Additionally, the problems encountered for the reported cases of the horizontal simulation are not present in this case, and for these particular experimental conditions, this model delivers better results. Similarly, the trend between these backscattering values appears to be consistent, as simulated values do not exceed measured values, and relative minima and maxima match also correctly (Figure 13(b)).

For this canopy type and observation conditions, this result shows that the simulated soil surface values are in accordance with the image measured backscattering coefficients, as the volumetric scattering contribution of the vegetation layer produces an additive effect on the returned signal and therefore the image measured values must be higher than those returned only by the soil surface. Consequently, this resulting dataset of the soil simulation process is definitely considered as the most suitable for characterizing the vegetation canopy, and will be then used for assessing the vegetation microwave scattering models selected for this study (Equations (4) and (6)).

Regarding the satisfactory results reached by this simulated vertical polarization state, they appear to be in agreement with the reported results given in [50], who point out that at this frequency band, $C-\sigma_{\mathrm{vv}}^{0}$ increases with the incidence angle. For this particular scene, the local incidence angle (θ_i_) at the image center is 38.50°, which is considered as a medium-high incidence angle. Moreover, concerning the behavior of the measured polarization state ( $C-\sigma_{\mathrm{vv}}^{0}$), In [51], authors considers that the sensitivity of this coefficient at this particular band, is strongly influenced by the planting pattern. In addition to the effect produced by the incidence angle, [52] states that the soil contribution for low foliar biomass levels (as those present in the study area), may be higher for the vertical polarization. Hence, these considerations might also justify the achieved results.

### 3.5. Characterization of the Vegetation Canopy

Once the simulation of the soil surface has been performed, the considered type of vegetation canopy is evaluated by means of the microwave scattering models introduced in Section 2.3.1, which are assessed in the following sections using a non linear regression based on the ‘Levenberg-Marquadt’ algorithm, where the convergence criterion for the sum of squares has been always set to 10^−8^. All statistical analyses have been carried out at a 5% significance level.

#### 3.5.1. RT Vegetation Scattering Models—Rayleigh Model

First, a Rayleigh Model (Equation (7)) has been applied, which uses as input the $C-\sigma_{\mathrm{vv}}^{0}$ values simulated by the Dubois Model, as well as their respective values measured on the RADARSAT 2 VV polarized channel. This model provides adjusted transmissivity (Γ) and albedo (ω) values, which are derived by the specified non-linear regression method.

**Figure 14.**
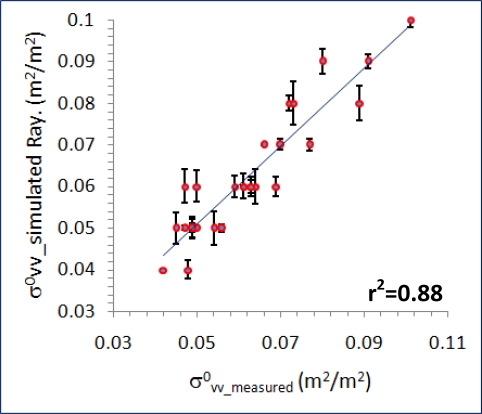
Agreement between measured and simulated values using Rayleigh Model for the vertical polarization state $\sigma_{\mathrm{vv}}^{0}$ (Error bars show individual error values).

Thus, the values obtained for these two variables are Γ = 0.91 and ω = 0.35, which indicate a very high transmissivity and a medium-low albedo or reflectivity. Figure 14 plots the best regression fit between measured and simulated canopy backscattering values, where a high coefficient of determination r^2^ = 0.88 (p-value = 4.23 × 10^−14^) is reached after six iterations of the model.

The results attained for these two variables may be in agreement with the considered canopy type, since olive groves are characterized by large gaps between rows of trees and by a low density of vegetation constituents, thus leading to this high transmissivity of the canopy. In turn, for reflectivity, which might be produced by volumetric scattering, its value is not as significant as it might occur for dense vegetation canopies. In this framework, it is not been possible to assess these results by means of direct methods, which in addition are spatially and temporally specific, and therefore they are only meaningful under these particular conditions. However, the knowledge of Γ and ω, and the corresponding system incidence angles, allow applying Equation (4), so that a raster layer of soil backscattering values can be produced for the observed vegetation canopies. In turn, this layer might be compared to a similar result obtained by inverting Equation (6), as both products represent the same phenomena, and are derived under the same experimental conditions. Instead, a different approach to verify the obtained values of transmissivity and albedo has been used, which is explained in the next section.

#### 3.5.2. RT Vegetation Scattering Models—Modified Xu and Steven Model

Usually, WCM models, using Leaf Area Index as a descriptor of the vegetation canopy, do not specify which of the two variables addressed in this paper, *i.e.*, true (LAI_true_) or effective LAI (LAI_eff_), must be considered. Subsequently, in this section, the performance and sensibility of this type of models to these two biophysical variables is assessed. For this purpose, the values for these two variables specified in Section 3.3 (Table 1) and corresponding to the different sampling units, are integrated into the modified version of the Xu and Steven Model (Equation (6)), together with the soil contribution simulated values ( $C-\sigma_{\mathrm{vv}\_\mathrm{Dubois}}^{0}$) referred in Section 3.4.

**Figure 15.**
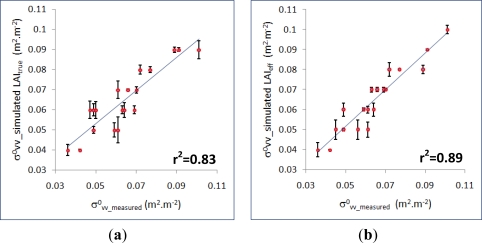
Regression analysis results between measured and simulated $\sigma_{\mathrm{vv}}^{0}$ values from the modified Xu and Steven model using, (**a**) True LAI, (**b**) Effective LAI. (Error bars show individual error values).

While there are some differences, these two reported cases are verified to converge properly. For LAI_true_ a coefficient of determination r^2^ = 0.83 (p-value = 1.56 × 10^−8^), has been reached after 14 iterations, whereas an r^2^ = 0.89 (p-value = 3.24 × 10^−10^) has been achieved for LAI_eff_ after seven iterations. The adjusted model parameters are A = 0.276 and B = 0.071 when true LAI is taken into account, while A = 0.405 and B = 0.115 when effective LAI is considered. These results indicate that in the first case (LAI_true_) the olive grove canopy is very thin with a very low attenuation, while in the second case (LAI_eff_) these values are higher, which appear to be more realistic and according to this vegetation cover. Moreover, as the coefficient of agreement is better for effective LAI, the characterization of this type of canopy by means of this biophysical variable is proven to be more acceptable, and might also imply that the grouping effect of the vegetation constituents (or their geometrical properties), is better taken into account by this type of microwave scattering models, as it has been suggested in [51,53,54]. Finally, these statements are also confirmed by means of the corresponding regression fits between simulated and measured backscattering coefficients depicted in Figure 15a and 15b, where it is noticed that the simulated $\sigma_{\mathrm{vv}}^{0}$ of the canopy using LAI_eff_ exhibits a higher dependency with the measured $\sigma_{\mathrm{vv}}^{0}$ (Figure 15(b)).

**Figure 16.**
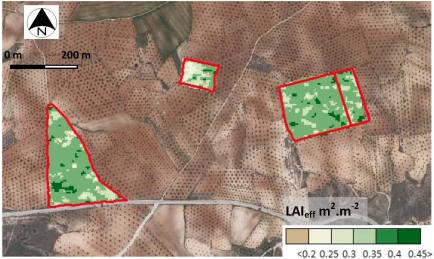
Effective LAI map derived by means of the proposed inversion procedure.

Once parameters A and B are then derived, Equation (6) can be used for different inversion purposes. In this work, the methodology introduced in Section 2.3.1 by means of Equation (7) is addressed, *i.e.*, extracting LAI values. Furthermore, our interest is focused in deriving LAI maps. This task can only be accomplished if a raster layer of soil backscattering values is supplied to Equation (7). This layer may be produced by inverting Equation (6). However it is preferred to follow a different issue using another source of information for this document, *i.e.*, the soil contribution derived by the Rayleigh method (Equation (4)). Moreover, when comparing the results with known LAI values, the correctness of the values for the transmissivity and albedo of the layer can also be confirmed, as it has been referred in Section 3.5.1. Hence, applying Equation (7) to the values of the RADARSAT 2 image using this proposed methodology with the set of derived parameters in this section, LAI values can be mapped for the considered sites of this study. Figure 16 shows such a map describing effective LAI values for the olive grove parcels containing the sampling units.

In a temporal framework, these documents might be systematically generated and used for environmental or agronomic monitoring, such as analysis and management of radiation and energy exchanges, as well as for subsequent measurement of the canopy photosynthesis [55]. In [56], it is pointed out that this variable and its distribution are fundamental for analyzing the canopy evapotranspiration, or for assessing the precipitation intercepted by leaves [57].

Finally, the results of this inversion process have been analyzed by comparing inverted values against field measured values. For this purpose, eleven LAI_eff_ and LAI_true_ field observations not included in the regression analysis have been used as reference values. These reference values are also distributed regularly throughout the 8 sampling units.

**Figure 17.**
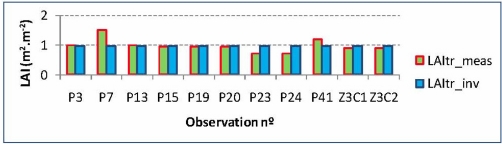
True LAI, measured *vs.* inverted values.

In Figure 17, inverted true LAI values (blue bars) are observed to be almost constant with values near to 1. It is found as well, that the modeling procedure for this particular variable does not account for variations within the canopy, e.g., observations P7, P23, P24 and P41, which means that the true LAI and the referred model may not be really suited for this purpose, as it has already been pointed out.

**Figure 18.**
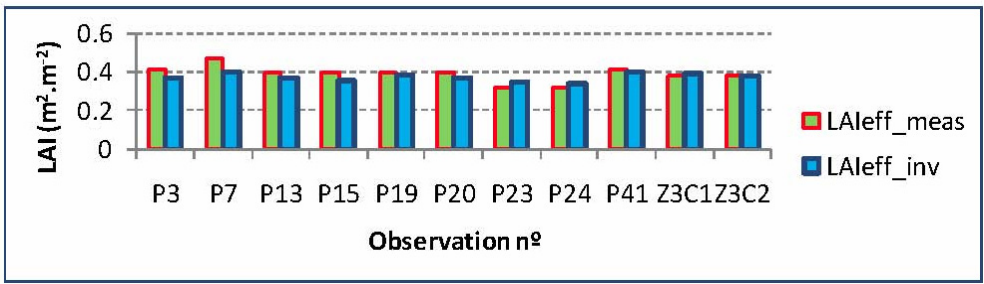
Effective LAI, measured *vs.* inverted values.

However, for effective LAI values, generally, the trend between measured and inverted values is verified to be quite satisfactory. However, some discrepancies may still be appreciated, as it might be the case for observation P7 (Figure 18), although for the remaining ten observations remain closer.

Therefore, this achieved result confirms again the good performance of effective LAI, which reveals to be a more pertinent variable compared to true LAI for characterizing the vegetation canopy by means of microwave scattering models. Furthermore, transmissivity and albedo values obtained by the Rayleigh model for these particular sites and at this specific time are proven to be also acceptable. Nevertheless, the statements and results issued from this work must still be verified using a higher number of observations, both for assessing the vegetation microwave scattering models and for analyzing the achieved results. As a final remark, in the case of effective LAI, using the full dataset of available observations for the adjustment of Equation (6), the coefficient of determination (r^2^) rises from 0.89 to 0.91, which may lead to a better definition of the model parameters A and B, and therefore deserve in an improvement of the inversion procedure.

## 4. Conclusions

In this paper, a methodology for characterizing vegetation canopies by means of microwave scattering models and optical means has been assessed. For this purpose, soil surface information has been acquired by classical methods, while the biophysical values of the vegetation layer have been derived by the hemispherical photography technique, which has not been extensively used for providing ancillary data in the field of radar systems applications.

For the soil dielectric surface simulation, the Dubois Model achieves the best results (r^2^ = 0.88). Given the soil surface conditions exhibited by the sampling units of this study and the properties of the radar system, the vertical polarization state at this particular band ( $C-\sigma_{\mathrm{vv}}^{0}$) appears to be the most appropriate for modeling the soil behavior for these types of soil surfaces, while the horizontal polarization state for these radar parameters does not deliver suitable results. In this sense, a full polarimetric mode might not be necessary for this type of landcover or canopies when the radar parameters, such as frequency and incidence angle, are well controlled. Nevertheless, the crosspolarized coefficient has not been assessed, and it might certainly supply valuable information. Therefore, a dual mode configuration might be the most appropriate.

Regarding the canopy biophysical variables, this study has distinguished between effective and true LAI’s. Accordingly, a modified version of the Xu and Steven Model has been applied and analyzed, which shows a good capability for assimilating true and effective LAI’s derived from the suggested technique. For this model, effective LAI is proven to return a better coefficient of determination (r^2^ = 0.89). Therefore, the knowledge of the variables related to canopy architecture improve the application of these type scattering models, and the sensor used for acquiring this information (hemispherical photography) provides suitable values for the involved biophysical variables. Furthermore, the convenience of an inversion procedure for generating thematic documents that represent the spatial distribution of LAI values is analyzed. For the observed canopies, under homogeneity conditions, the discrepancies between inverted and measured values are minimal, which confirms that the behavior of this type of model is appropriate under certain conditions of homogeneity.

As a concluding remark, this work proves that the suggested approach has the potential of retrieving biophysical parameters from SAR data. Nevertheless, additional efforts must be still done in order to extend this methodology to wider areas, other vegetation canopies typologies and biophysical variables. Furthermore, these new studies must be performed in a multitemporal framework, with a radar multifrequency dataset, as well as with other measuring sensors for deriving common sets of values for physical and biophysical variables, so that the results can in turn be compared and assessed.

## Figures and Tables

**Table 1.** Summary of Gap Fraction and LAI values for the Sampling Units (SU).

**SITE/SU#/VC#**		**LAI_eff_ (m^2^·m^−2^)**	**LAI_eff 57°_ (m^2^·m^−2^)**	**LAI (m^2^·m^−2^)**	**σ_Po_ (0°)**	**σ_Po_ (57°5)**	**σ_Po_ (60°)**	**Longitude (λ)**	**Latitude (φ)**
1	SU1/VC1	0.41	0.22	1.5	0.05	0.08	0.10	3°28′55″W	40°13′47″N
	SU2/VC2	0.47	0.34	1	0.02	0.12	0.12	3°28′56″W	40°13′44″N
	SU3/VC3	0.40	0.19	1	0.03	0.07	0.12	3°28′58″W	40°13′45″N
	SU4/VC4	0.40	0.26	0.94	0.03	0.10	0.12	3°28′58″W	40°13′47″N
									
2	SU5/VC5	0.32	0.27	0.71	0.02	0.08	0.10	3°28′51″W	40°13′45″N
									
3	SU6/VC6	0.26	0.15	0.47	0.06	0.07	0.10	3°29′34″W	40°13′44″N
	SU7/VC7	0.28	0.22	0.54	0.03	0.10	0.10	3°29′31″W	40°13′39″N
									
4	SU8/VC8	0.41	0.22	1.2	0.02	0.08	0.10	3°29′13″W	40°13′49″N
